# Quantifying the benefits of reducing synthetic nitrogen application policy on ecosystem carbon sequestration and biodiversity

**DOI:** 10.1038/s41598-022-24794-2

**Published:** 2022-12-01

**Authors:** N. Devaraju, Rémi Prudhomme, Anna Lungarska, Xuhui Wang, Zun Yin, Nathalie de Noblet-Ducoudré, Raja Chakir, Pierre-Alain Jayet, Thierry Brunelle, Nicolas Viovy, Adriana De Palma, Ricardo Gonzalez, Philippe Ciais

**Affiliations:** 1 Laboratoire des Sciences du Climat et de l`Environnement LSCE/IPSL, Unité Mixte CEA-CNRS-UVSQ, Université Paris-Saclay, 91191 Gif-Sur-Yvette, France; 2 CIRAD, UMR CIRED, 94736 Nogent-Sur-Marne, France; 3 US ODR, INRAE, 31326 Castanet-Tolosan, France; 4 College of Urban and Environmental Sciences, Peking University, Beijing, China; 5 Université Paris-Saclay, INRAE, AgroParisTech, PSAE, 91120 Palaiseau, France; 6 Department of Life Sciences, Natural History Museum, Cromwell Road, London, SW7 5BD UK; 7 Department of Life Sciences, Imperial College London, Silwood Park, Berkshire, SL5 7PY UK; 8 Present Address: Services for Computational Research, CSC - IT Center for Science, 02101 Espoo, Finland

**Keywords:** Biogeochemistry, Climate sciences, Ecology, Environmental sciences

## Abstract

Synthetic Nitrogen (N) usage in agriculture has greatly increased food supply over the past century. However, the intensive use of N fertilizer is nevertheless the source of numerous environmental issues and remains a major challenge for policymakers to understand, measure, and quantify the interactions and trade-offs between ecosystem carbon and terrestrial biodiversity loss. In this study, we investigate the impacts of a public policy scenario that aims to halve N fertilizer application across European Union (EU) agriculture on both carbon (C) sequestration and biodiversity changes. We quantify the impacts by integrating two economic models with an agricultural land surface model and a terrestrial biodiversity model (that uses data from a range of taxonomic groups, including plants, fungi, vertebrates and invertebrates). Here, we show that the two economic scenarios lead to different outcomes in terms of C sequestration potential and biodiversity. Land abandonment associated with increased fertilizer price scenario facilitates higher C sequestration in soils (+ 1014 MtC) and similar species richness levels (+ 1.9%) at the EU scale. On the other hand, the more extensive crop production scenario is associated with lower C sequestration potential in soils (− 97 MtC) and similar species richness levels (− 0.4%) because of a lower area of grazing land. Our results therefore highlight the complexity of the environmental consequences of a nitrogen reduction policy, which will depend fundamentally on how the economic models used to project consequences.

## Introduction

Over the last century, agricultural production and a growing human population have become heavily dependent on the use of Nitrogen (N) fertilizers^1–3^. For instance, in 2017, 11.6 million tons of N fertilizer were used in European Union (EU) agriculture, an increase of 8% since 2007 which led to the harvest of 310 million tons of cereals (source: EUROSTAT, EU 2018). The contribution of N fertilizer application to increasing plant productivity and consequent changes in land-use and agricultural yields has long been recognized^1,4,5^. However, the negative impacts of N fertilizer on the environment in Europe are also visible and are on average more pronounced than in the rest of the world^6^. That is because much of the N used in agriculture is emitted to the atmosphere and leaches into the groundwater, which causes a cascade of environmental problems (e.g. groundwater contamination, and soil acidification^3^). Europe is an N hotspot in the world with high N export along rivers to the coast, with emissions of nitric oxides, nitric acid, and nitrate-containing particles accounting 10% of global N_2_O emissions^6^.

N fertilizer also has numerous impacts on agricultural soils, including changes in soil structure, soil nitrogen and carbon cycles^1,7,8^. The historical and ongoing increase in agricultural production has contributed and continues to contribute to land-use change, which in turn continues to significantly increase the atmospheric carbon dioxide (CO_2_) concentration. Globally, agricultural production contributes ~ 24% of greenhouse gas emissions^9–12^. Across Europe, more than 50% of the original forest has been cleared to make way for croplands and pasturelands, and as a source of fuelwood and construction materials^13–15^. Such intensive agriculture across Europe may have decreased soil carbon stocks in many regions and contributed to increased atmospheric CO_2_ concentration^16,17^ but also allowed a widespread increase in agricultural yields has been observed all over Europe.

Fertilizer addition and agricultural intensification have also had negative consequences for ecosystem function and biodiversity^13,18–21^. An increase in fertilizers often results in a decline in plant species richness^20,21^ and changes in community structure and functional composition^1,20,22^. Newbold et al.^19^, although they do not explicitly consider the effect of N fertilizer application but a suite of management practices, showed that land-use is associated with species richness to reduce by an average of 76.5%, total abundance by 39.5%, and rarefaction-based richness by 40.3%. In the recent global report on biodiversity and ecosystem services^23^, IPBES (Intergovernmental Science-Policy Platform on Biodiversity and Ecosystem Services) sounds the alert about the severity of biodiversity degradation and about the importance of taking biodiversity into account in environmental impact assessments of land use policies in order to halt this massive decline.

From the aforementioned discussion, it is clear that though global agricultural productivity is heavily dependent on the use of N fertilizers, many studies^1,24–28^ demonstrate that the long-term addition of fertilizers can also strongly affect ecosystem carbon and biodiversity. The objective of this study is therefore to analyze the effects of a public policy scenario aiming at halving the use of N fertilizers on key environmental variables—biodiversity indicators and carbon (C) sequestration—using a set of land-use, vegetation, and biodiversity models. One major challenge is that each change affecting one of the environmental variables results from a complex mechanism implying a change in intensity versus change in area. This study attempts to disentangle this mechanism, by separately evaluating the effect of each driving factor (intensity and area) on a given environmental variable (biodiversity and C sequestration). Further, our ultimate focus is to investigate the N fertilizer induced changes in land use and its impacts.

At the scale of the EU, specifically in this study, we focus on analyzing changes in net primary productivity (NPP), carbon in biomass and soil, the abundance-based biodiversity intactness index (BII), and species richness (SR) due to a 50% reduction in N-fertilizer induced land use and land cover changes. The next section, materials and methods, briefly describes the modeling framework and the simulations performed. The results section then quantifies the changes in NPP, biomass carbon and soil carbon, and biodiversity indicators. The last section includes a discussion of the results and our conclusions.

## Materials and methods

### Overview of modeling framework

We have used a range of econometric, economic, and agricultural land surface models to analyze the factors driving land-use change in order to assess their ecological, agricultural, climatic and economic impacts. These multi-scale models differ in their methodologies, scale of interest, and resolution, but they are very complementary and could provide a unique opportunity to analyze public policy scenario effects on land-use and resulting changes in ecosystem carbon and biodiversity.

**Figure 1:**
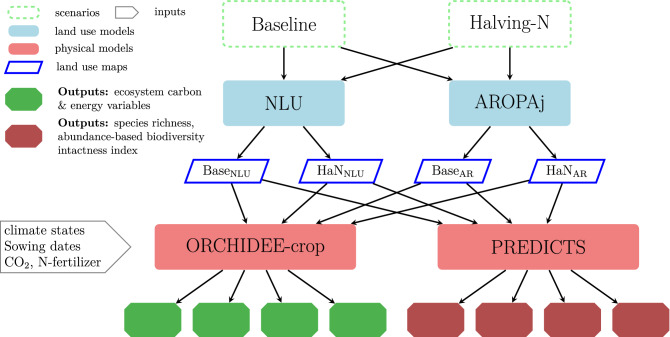
Schematic diagram illustrating the coupling of multi-scale land-use models. The multi-scale models coupled in this study are econometric, and economic models (NLU and AROPAj), an agricultural land surface model (ORCHIDEE-crop), and a biodiversity model (PREDICTS). Coupling means, we use the output of one model as an input to other models. In addition, we have performed one-way coupling and there is no two-way interaction between models. Each economic model generates two land-use maps corresponding to *Baseline* and *Halving-N* scenario which are inputs (2 from NLU and 2 from AROPAj) to ORCHIDEE-crop and PREDICTS. The ecosystem carbon (C) sequestration is simulated by ORCHIDEE-crop and biodiversity indicators are simulated by PREDICTS model. The abbreviations ‘Base_NLU_’ and ‘HaN_NLU_’ means *Baseline* and *Halving-N* land-use map generated by NLU model. The abbreviations ‘Base_AR_’ and ‘HaN_AR_’ means *Baseline* and *Halving-N* land-use map generated by AROPAj model.

Among these models, the economic land use model Nexus Land Use (NLU)^29,30^ and the agricultural supply-side model Agriculture, Recomposition de l’Offre et Politique Agricole (AROPAj)^31^ coupled with a spatial econometric model^32^ have allowed us to estimate the impact on EU land-use of a scenario involving a 50% reduction in N synthetic fertilizers compared to a baseline scenario. In the present study, we use these land-use scenarios to force ORCHIDEE-crop (Organising Carbon and Hydrology in Dynamic Ecosystems), an agricultural land surface model^16,33^ and Projecting Responses of Ecological Diversity in Changing Terrestrial Systems (PREDICTS)^34^, a biodiversity model to simulate, respectively, ecosystem C and biodiversity changes across the EU covering the domain 35.25°N and 69.25°N in latitude and 9.25°W and 34.25°W in longitude. The schematic (Fig. 1) provides a brief overview of the modelling framework applied in this study.

In order to link the land use output data from the AROPAj and NLU models with the ORCHIDEE-crop and PREDICTS models, the first step is to match land uses and crops between the models (see Table 1). AROPAj and NLU crops are classified into ORCHIDEE-crop plant functional types (PFTs): C3 winter and summer crops, C4 summer crop and C3/C4 natural grass (see “Model descriptions” section for a detailed description of ORCHIDEE-crop PFTs). The AROPAj and NLU crops are also classified into the PREDICTS crop types: annual, perennial, N-fixing. The AROPAj and NLU "rangeland" and "pasture" categories are found in PREDICTS but in ORCHIDEE-crop they are considered to fall within the C3 natural grassland PFT. Finally, NLU and AROPAJ forest and other natural areas are classified as "primary" natural areas (with low anthropogenic use) or "secondary" (intermediate to high anthropogenic environmental use) according to the land use map of these areas^35^. For ORCHIDEE-crop, they are classified as natural forest PFTs. Note that the fallow areas described in AROPAj that are part of crops are classified as "grass" PFT in ORCHIDEE-crop and as "minimum" intensity annual crops in PREDICTS.

**Table 1:** Table of correspondences between the land uses and crops represented in the AROPAJ/NLU and ORCHIDEE models and PREDICTS.

	NLU																				
	Cassava	Fieldpea	Groundnut	Maize	Millet	Rapeseed	Rice	Soybean	Sugarbeet	Sunflower	Wheat	Other	Pasture	Forest	Urban						
Corresponding land-use in PREDICTS	Annual	C3Nfx	C3Nfx	Annual	Annual	Annual	Annual	C3Nfx	Annual	Annual	Annual	Annual/Perennial	Pasture/rangeland	Primary/Secondary	Urban						
Corresponding land-use in ORCHIDEE	C3/C4 natural grass	C3 summer crop	C3 summer crop	C4 summer crop	C3 summer crop	C3 winter crop	C3 summer crop	C3 summer crop	C3 summer crop	C3 summer crop	C3 winter crop	Bare soil	C3/C4 natural grass	Temperate and boreal needle leaf, broadleaf, evergreen, summer green trees	Bare soil						

	AROPAJ																				
	Pasture	Rangeland	Urban	Other ecosystem	Forest	Durum wheat	Tender wheat	Winter barley	Spring barley	Oats	Other cereals	Rice	Maize	Fallow	Beetroot	Rapeseed	Sunflower	Soybean	Other legumes	Potato	Perennial
Corresponding land-use in PREDICTS	Past	Range	Urban	Primary/Secondary*	Primary/Secondary*	Ann	Ann	Ann	Ann	Ann	Ann	Ann	Ann	Ann	Ann	Ann	Ann	C3Nfx	C3Nfx	Ann	Perennial
Corresponding land-use in ORCHIDEE	C3/C4natural grass	C3/C4 natural grass	Baresoil	Baresoil	Temperate and boreal needle leaf, broadleaf, evergreen, summer green trees		C3 winter crop	C3 winter crop	C3 summer crop	C3 summer crop	C3 summer crop	C3 summer crop	C4 summer crop	C3/C4 natural grass	C3 natural grass	C3 winter crop	C3 summer crop	C3 summer crop	C3 natural grass	C3 natural grass	–

Crops or land uses in NLU or AROPAj that are found in more than one land use in PREDICTS or ORCHIDEE are allocated between land-uses according to the rules described in “Overview of modeling framework” section.

The land-use and land cover changes described in the following sub-section are used as inputs to ORCHIDEE-crop and PREDICTS from both the NLU and AROPAj models’ output.

### Land-use change scenarios

Land-use changes in the EU are simulated for the present day using two scenarios: (1) a business as usual scenario (*Baseline*) and (2) a scenario involving a policy to reduce mineral nitrogen use by 50% from the Baseline (*Halving-N*). The land-use changes in *Halving-N* and *Baseline* are computed by both NLU and AROPAj models. In the latter model, the computed land-use changes result from coupling between AROPAj and a spatial econometric model. Since there are differences in the nature of the models (supply-side model versus partial equilibrium model) and their underlying data, the *Baseline* scenarios in the NLU and AROPAj frameworks are different. A detailed description of the differences and a discussion of their implications on the production and area of different land-uses is provided in Lungarska et al.^36^. EU plant production is 370 and 383 MtDM (Million tons of Dry Matter) respectively based on the application of 12 TgN (Tera grams) of N fertilizer in AROPAj and NLU. Crops, grasslands, and forests cover respectively, 116, 57 and 234 Mha in NLU and respectively 94 (including fallow land), 38 and 142 Mha in AROPAj. In AROPAj and NLU, the 50% N reduction is achieved indirectly by increasing the N input price from present-day figures^36^.

The land-use changes output from AROPAj and NLU are supplied as inputs to the ORCHIDEE-crop and PREDICTS models. The land-use changes are matched with corresponding plant functional types (PFTs) in ORCHIDEE-crop and land-uses in PREDICTS (see Table 1). “Model descriptions” section provides a detailed description of the ORCHIDEE-crop and PREDICTS models.

### Model descriptions

Here, we describe the ORCHIDEE-crop and PREDICTS models that quantify the impacts of halving N fertilizer consumption in the EU. Table 2 presents a brief overview of the two models.

**Table 2:** Overview of the ORCHIDEE-crop and PREDICTS models input and output.

Models	Input	Output	Resolution
ORCHIDEE-crop- Agricultural land surface model^40^	Meteorological forcing (Air temperature, specific humidity, incoming shortwave and longwave radiation, rainfall), land use change scenarios, CO_2_, N fertilizers etc.	Energy and water balance, ecosystem carbon, CO_2_ emissions, productivity etc.	50 km × 50 km
PREDICTS-biodiversity model^34^	Land use change scenarios. *No link between biodiversity and climate in this model*	Species richness (SR), Biodiversity intactness Index (BII)	50 km × 50 km

*A detailed description of ORCHIDEE-crop*: This model is a process-based agricultural land surface model that integrates crop-specific phenology based on Simulateur mulTidisciplinaire pour les Cultures Standard (STICS)^37,38^. Carbon allocation is based on the plant-based hybrid model from the original ORCHIDEE allocation scheme^39^ and a crop specific formulation of STICS providing leaf, root, and shoot biomass, grain maturity time, litter production, and litter and soil carbon decomposition. The harvest date is calculated after grains reach maturity^40^. The ORCHIDEE-crop model has no explicit nitrogen cycle but accounts empirically for the effect of N fertilization by increasing the maximum Rubisco- and light-limited leaf photosynthetic rates as a function of the amount of N applied, using a Michaelis–Menten function^40^. Also, ORCHIDEE-crop is calibrated against observations, which showed a good match between modeled observed aboveground biomass, crop yield, and daily carbon^40^. This version of the model currently uses three crop PFTs: C3 winter, C3 summer and C4 summer. Forests are classified as Broadleaf, Needle leaf, Deciduous, Temperate and Boreal. Up to 11 non-cropland vegetation types can co-exist with crops on a grid point of the model, according to prescribed land cover information. A gridded simulation of ORCHIDEE-crop requires 30-min time step meteorological forcing (air temperature, specific humidity, incoming shortwave and longwave radiation, rainfall), which can be interpolated in time from gridded climate analysis data or atmospheric models. In this study, this model is used to quantify the ecosystem C variables.

*A detailed description of PREDICTS*: The PREDICTS database was collated by searching the published literature for studies where terrestrial biodiversity (including plants, fungi, vertebrates, and invertebrates) was sampled using consistent methods across multiple sites, which vary in the pressures faced. The land use and intensity of each site have been assessed and categorized in a consistent way^41–43^. Authors of studies were contacted to ask for the raw biodiversity data where this was not already available^41,42^. Most records in the PREDICTS database refer to the number of individuals of a species at a site; this makes it possible to compute a range of biodiversity indices. To estimate biodiversity responses to human impacts across such a global and heterogeneous dataset, linear mixed-effects models are used; random intercepts account for differences in biogeographic factors, sampling methodology and taxonomic focus, and the spatial layout of sites within studies. Using the PREDICTS database to assess the impact of human pressures on biodiversity assumes that space-for-time substitution is valid^44^; it assumes that the sites have reached equilibrium and so the impact of pressures on biodiversity over time can be observed across space and that the relationship between biodiversity and drivers do not vary over time.

SR is calculated as the number of species at each site; it is a widely used measure of biodiversity and is both simple and intuitive. Responses of SR to land use and intensity were modelled using generalized linear mixed effects models and with a Poisson error structure; an observation-level random effect was included to account for overdispersion^45^. This model is then used to project SR in each grid of a 0.5° map and expressed as a percentage of the SR level in primary vegetation from land use harmonization map^35^.

To estimate BII change with land use and intensity, two models are required. Total abundance was first calculated as the sum of all individuals at each site; it was then rescaled within the study (so that the maximum within a study is 1) and was square-root transformed before modelling as a function of land use and intensity, to account for non-normality of the model residuals (a Poisson error structure could not be used as abundance data can include non-integer data e.g. densities). Inclusion of a random slope for land use within the study was supported (based on Akaike’s Information Criterion). Compositional similarity was then calculated as the asymmetric Jaccard index, comparing each baseline site (primary vegetation) with all other sites, and logit transformed with an adjustment of 0.01 (to account for non-normality of the model residuals). Compositional similarity was then modelled as a function of land use and intensity (coarsened so that only perennial crops were allowed to differ across intensities), including the environmental and geographic distance between sites as control variables, whose effects were permitted to differ among land use and intensity levels (these variables were cube-root and log-transformed respectively to improve residual distribution). To calculate BII, total abundance (expressed as a percentage of their level in primary vegetation) and compositional similarity (expressed as a percentage of their level in primary vegetation)^46^ are projected for each grid of a 0.5° map; these two maps are then multiplied to give abundance-based BII^19^. The PREDICTS models include different levels of management (intensive, light or minimal) and different types of land cover (forest, pasture, rangeland, annual cropland, perennial cropland, and urban zones). The coefficients of these mixed-effect models and a detailed description of the link between the PREDICTS models and NLU are available in Prudhomme et al.^46^. The spatial predictions of biodiversity were computed using a python pipeline, which was developed specifically for the PREDICTS project (https://github.com/ricardog/raster-project).

In our modeling framework, the impact of halving N fertilizer goes through two steps: (i) we calculate the effect of this reduction of N fertilizer on agricultural yield, and (ii) calculate the effect of the yield reduction on biodiversity. By keeping yield as a proxy of agricultural land use intensification as proposed in Prudhomme et al.^46^, we include not only the direct effect of the reduction of N fertilizer on biodiversity but also the effects correlated to this reduction of N fertilizer such as the reduction of other chemical inputs (P and K fertilizers and pesticides). While the effect of the change in N fertilization on yield is calculated by the classical concave production function in agronomy^29^, the effect of the change in yield is calculated by coupling the NLU land use model and the PREDICTS biodiversity model^46^. For each category of crops (annual, perennial, leguminous), the coupling consists of estimating (using a Generalized Additive Model [GAM]) the share of each intensity class (minimum, light, intense) as a function of the average calorie yield based on the average crop yield maps from a plant growth model. The maps describing the share of land use intensities are from Newbold et al.^19^ Similarly for pasture, the share of each intensity class (light, intense) is estimated with the help of a GAM as a function of ruminant density.

### Simulations

Our experimental design focuses on assessing the effects of a 50% reduction in present-day N fertilizer use levels across the EU. The choice of halving N fertilizer in EU agriculture is related to the “Farm to Fork” strategy, which puts forward the ambition for 2030 to reduce nutrient losses to the environment from both organic and mineral fertilizers by at least 50%. The results from NLU (and its nitrogen balance module) show that this level of reduction corresponds to a 50% reduction in nutrient losses (nitrogen and phosphorus) aimed by the Farm to Fork strategy as a part of the European Green Deal. AROPAj models exclusively the EU countries (in 2012, there were 28 member states) while NLU simulations cover the EU and the rest of the world (EU being a part of the European region as represented by the model). However, the N reduction policy implemented in the EU alone and the comparison of the results conducted only for the EU. All EU member states are considered but for some of them we present results. A total of four simulations corresponding to four land-use maps (two from AROPAj and two from NLU, see Fig. 1) are performed in the ORCHIDEE-crop model and also in the PREDICTS model. In addition to changes in the area of different land-uses, changes in mineral N input are accounted for in both models. However, changes in organic N input and crop rotations are not accounted for. In ORCHIDEE-crop 55% of the carbon harvested from croplands is exported but the remaining residues are returned to the soils.

*ORCHIDEE-crop simulation details*: the model simulations are performed over a domain covering the EU. Four idealized simulations are carried out using the ORCHIDEE-crop model by forcing present-day meteorological data (2006–2010), levels of N fertilizer (150 KgN/ha) and atmospheric CO_2_ concentration (385 ppm). The four simulations include *Halving-N* and *Baseline* corresponding to AROPAj and NLU land-use scenarios (two ORCHIDEE-crop simulations per economic model). All four simulations start from the year 2010 climate and carbon cycle conditions with a recycled climate (2006–2010) for 150 years. For the year 2010, climate and carbon cycle conditions are obtained from the output of historical simulations. Historical simulations from the year 1901 to the year 2010 are performed for both AROPAj and NLU *Baseline* scenario land-use land cover maps. In addition, these historical simulations started from an equilibrium state of soil carbon, energy and water cycle variables corresponding to the year 1901. The 1901 equilibrium state is determined by running a 350-year spin-up simulation corresponding to a recycled climate (1901–1910). The observation-based climate forcing data from the Global Soil Wetness Project was only available starting from the year 1901. The drift in soil carbon over the last 100 years of the 350-year simulations is less than 1%. The equilibrium state simulations corresponding to the year 1901 were necessary to have stabilized biophysical and ecosystem C variables across the EU. Other forcing variables, e.g. atmospheric CO_2_ concentration (296.57 ppm), N-fertilization rate (32 KgN/ha), harvest index (0.25), and also the phenology parameters for short-cycle variety winter and summer crops^16^ corresponding to the year 1901 were prescribed.

*PREDICTS simulation details*: the PREDICTS model represents changes in broad-sense biodiversity in different land-uses and intensities of land-use relative to a reference land-use (as the biodiversity metrics assessed include all terrestrial biodiversity for which data are present in the PREDICTS database including plants, fungi, vertebrates and invertebrates). Here the reference ecosystem is a primary natural ecosystem. Biodiversity changes are then reported as a percentage by dividing the obtained biodiversity levels by the level of biodiversity present in the primary natural ecosystem. This simulation is performed for each grid point on a map of the EU for land-use scenarios corresponding to *Baseline* and *Halving-N* for both economic models, AROPAj and NLU (Fig. 1).

### Breakdown method for biodiversity and carbon changes

The *Halving-N* and *Baseline* scenarios provide contrasted land-use maps according to the assumptions of economic and land-use models^36^. This results in different plant and animal production, and different land-uses at the European scale in each model. A price shock on inputs, as represented in the *Halving-N* scenario compared to the *Baseline* scenario, can induce (1) a spatial reallocation of production or (2) production changes^47^. Here, we separate out the effects of these two mechanisms on biodiversity (species richness) and carbon indicators (NPP and soil carbon) by decomposing the overall environmental differences between the *Halving-N* and the *Baseline* scenarios. The breakdown is not possible for the BII indicator because this indicator is the product of two indicators: abundance and a similarity indicator of ecological communities.

First, we breakdown the carbon and biodiversity differences by land-use type. The breakdown for carbon is straightforward because the carbon changes are computed for each land-use. The biodiversity changes associated with each land-use are computed by setting no changes in the other PREDICTS model land-uses. The sum of the biodiversity changes for each land-use is thus equal to the overall change in biodiversity.

For each land-use *i* (forest, grassland and cropland), we separate out the carbon and biodiversity differences between the *Halving-N* and the *Baseline* scenarios into two effects in accordance with Eq. (1): (i) the carbon and biodiversity difference associated with the area difference—called “Area effect”, and (ii) the carbon and biodiversity difference associated with the difference in biodiversity and carbon sequestration per unit area—called “Intensity effect”. The “Area effect” corresponds to the change in carbon sequestration and biodiversity associated with a change in the land-use area. For example, a reduction in grassland area leads to reduction in the C sequestration and biodiversity associated with this area. The “Intensity effect” corresponds to a change in the C sequestration and biodiversity per unit area. For example, a reallocation of production toward places with high soil C content leads to an increase in the carbon stock per hectare or an increase in crop yield leads to a reduction in the biodiversity per unit of cropland. Thus, the “Intensity effect” corresponds to the effect of a production reallocation on C sequestration, and the effect of land-use intensity on biodiversity.

We use the Logarithmic Mean Division Index (LMDI) method, which breaks down the target values into several main influencing factors based on mathematical identity transformation^48^ as follows.

1 $$\Delta {E}_{i}=\Delta {E}_{i}^{A}+\Delta {E}_{i}^{I}$$

$\Delta {E}_{i}$ is the difference in the environmental indicator between the *Halving-N* and the *Baseline* scenarios. Superscript ‘*A*’ denotes area effect and ‘*I*’ denotes intensity effect. Subscript ‘*i*’ denotes different land-use (e.g. forests, grassland, cropland etc.). $\Delta {E}_{i}^{A}$ is the difference in the environmental indicator between the *Halving-N* and the *Baseline* scenarios associated with the difference in area. $\Delta {E}_{i}^{I}$ is the difference between the *Halving-N* and the *Baseline* scenarios associated with the different intensity per unit of area of the environmental indicator.

2 $$\Delta {E}_{i}^{A}=\frac{{E}_{i}^{hN}-{E}_{i}^{b}}{ln({E}_{i}^{hN})-{ln(E}_{i}^{b})}\times ln\left(\frac{{A}_{i}^{hN}}{{A}_{i}^{b}}\right)$$

${E}_{i}^{hN}$ is the level of the environmental indicator in the *Halving-N* (superscript *hN*) scenario. ${E}_{i}^{b}$ is the level of the environmental indicator in the *Baseline* (superscript *b*). ${A}_{i}^{hN}$ is the area of land-use *i* in the *Halving-N* scenario. ${A}_{i}^{b}$ is the area of land-use *i* in the *Baseline*

3 $$\Delta {E}_{i}^{I}=\frac{{E}_{i}^{hN}-{E}_{i}^{b}}{ln({E}_{i}^{hN})-ln({E}_{i}^{b})}\times ln\left(\frac{{e}_{i}^{hN}}{{e}_{i}^{b}}\right)$$

Equation (3) is same as Eq. (2) but for the intensity of the environmental indicator ${e}_{i}$.

The breakdown of the differences in the environmental indicators is performed between the *Halving-N* scenario and the *Baseline*. A positive variation ($\Delta {E}_{i}>0$) indicates a higher environmental indicator in the *Halving-N* scenario compared to the *Baseline* without implying any temporal variation since the scenarios compare the environmental indicator status in 2012 in the AROPAj and in the NLU land-uses. Conversely, a negative variation ($\Delta {E}_{i}<0$) indicates a lower environmental indicator in the *Halving-N* scenario compared to the *Baseline*.

## Results

### Changes in NPP

**Figure 2:**
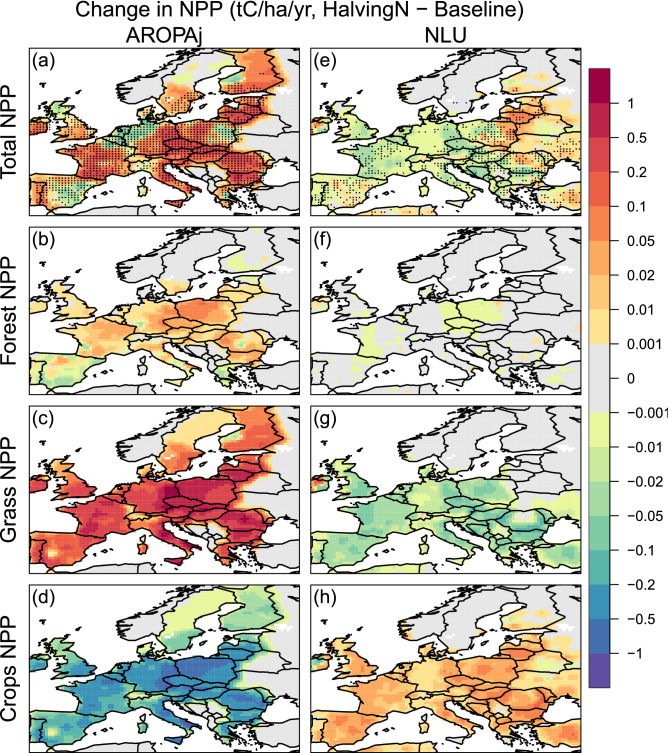
ORCHIDEE-crop model simulated annual mean change in (**a**, **e**) total NPP (tC ha^−1^ year^−1^), (**b**, **f**) Forest NPP, (**c**, **g**) Grass and Pasture NPP, and (**d**, **h**) Crop NPP due to 50% reduction in N fertilizer. The mean changes are computed using the last 50-years’ means of the 100-year simulations. The change in NPP shown here is the weighted sum across all PFTs. Stippled areas are regions where changes are statistically significant at the 95% confidence level. Significance level is estimated using a Student’s t-test with a sample of 50 annual mean differences and standard error corrected for temporal serial correlation. This figure is created using software R version 3.6.0 (https://www.r-project.org).

The simulated spatial changes in annual mean NPP between the *Halving-N* and *Baseline* experiments for both AROPAj and NLU land-use change scenarios are quantified (Fig. 2). Overall, a reduction in N fertilizer across the EU contributes to a significant increase in total net primary production of 38.45 million tons of C per year (MtCyr^−1^) as simulated by ORCHIDEE-crop for the AROPAj scenario (Tables 3, 4). Increase in forests, pastures and grasslands or other herbaceous vegetation (Fig. S1a, c) contributes to this total NPP increase. Spatially the increase in NPP is simulated over many EU countries (Fig. 2a–c). A significant increase is simulated for the United Kingdom (0.42 MtCyr^−1^), France (5.76 MtCyr^−1^), Italy (3.44 MtCyr^−1^), some parts of Germany (3.20 MtCyr^−1^), Poland (1.79 MtCyr^−1^), the Czech Republic (1.68 MtCyr^−1^) and Austria (1.14 MtCyr^−1^). Over some regions, total NPP significantly decreases (Fig. 2a). For instance, the decrease in parts of Spain, Belgium, and the Netherlands is due to a decrease in productive croplands NPP (Fig. 2d) and forests NPP (Fig. 2b).

**Table 3:** Annual change in total Net Primary Production (MtC/yr), Soil carbon (MtC), BII (%) and SR (%) between *Halving-N* and *Baseline* simulations across the EU along with selected EU countries. The changes are computed from the last 50 years’ annual averages of the 150-year simulation.

Country	Change in net primary production (MtC/yr)		Change in soil carbon (MtC)		Change in BII (%)		Change in SR (%)	
	AROPAj	NLU	AROPAj	NLU	AROPAj	NLU	AROPAj	NLU
Europe	+ 38.45	− 2.71	+ 1014.13	− 97.11	2	1.1	1.9	− 0.4
Austria (AUT)	+ 1.14	− 0.15	+ 24.28	− 2.99	1.6	1.5	1.9	− 0.7
Belgium (BEL)	− 0.09	− 0.12	+ 5.00	− 1.63	2.1	0.9	2.3	− 0.3
Czech Republic (CZE)	+ 1.68	− 0.21	+ 36.91	− 3.60	3.1	0.5	2.6	− 0.2
Germany (DEU)	+ 3.20	− 0.41	+ 120.23	− 6.82	1.8	0.4	3.0	− 0.2
Spain (ESP)	+ 0.44	− 0.12	+ 51.56	− 12.64	1.7	4.2	1.9	− 1.4
Finland (FIN)	+ 2.06	+ 0.15	+ 29.18	+ 0.11	0.4	0	0.6	0
France (FRA)	+ 5.76	− 0.83	+ 122.86	− 14.22	2.8	0.6	2.8	− 0.3
United Kingdom (GBR)	+ 0.42	− 0.19	+ 24.11	− 4.96	2.6	2.0	1.9	− 0.8
Hungary (HUN)	+ 1.49	− 0.08	+ 31.41	− 4.10	2.6	1.5	2.4	− 0.7
Italy (ITA)	+ 3.44	− 0.18	+ 103.71	− 5.19	4.3	0.7	3.6	− 0.3
Netherlands (NLD)	− 0.16	− 0.03	+ 2.62	− 0.48	6.1	1.1	3.2	− 0.4
Poland (POL)	+ 1.79	− 0.16	+ 137.38	− 7.83	3.3	0.7	3.0	− 0.3
Romania (ROU)	+ 4.75	− 0.08	+ 108.99	− 8.73	3.2	2.5	3.2	− 1.1
Sweden (SWE)	+ 0.67	− 0.003	+ 13.13	− 0.03	0.4	0	0.3	0

**Table 4:** Annual mean biodiversity and carbon values across the European Union (EU).

	AROPAj		NLU	
	Baseline	Halving-N	Baseline	Halving-N
BII (%)	78.7	80.1	81	82
SR (%)	83.7	85.7	81	80.5
NPP (MtC/yr)	2102.84	2141.29	2020.10	2017.39
Soil carbon (MtC)	20,706.55	21,720.68	19,487.78	19,390.67
Biomass carbon (MtC)	11,300.24	11,605.51	15,541.46	15,514.08

With the NLU land-use change scenario, the average simulated response in total NPP (Fig. 2e) contrasts with the results of the AROPAj land-use scenario (Fig. 2a). Forest area do not change in NLU (Fig. S1b) and hence no change in forest NPP (Fig. 2f). However, a reduction in N fertilizer causes a decrease in grazing land NPP in most parts of Europe (Fig. 2g). The total NPP production decrease across the EU is 2.71 MtCyr^−1^ (Tables 3, 4). This decrease is significant in France (− 0.83 MtCyr^−1^), Germany (− 0.41 MtCyr^−1^), the United Kingdom (− 0.19 MtCyr^−1^), and Italy (− 0.18 MtCyr^−1^) compared to the other EU countries (Fig. 2e, Table 3). The NPP decrease is mainly due to the loss of herbaceous vegetation (Fig. 2g) and grazing land being converted to cropland (Fig. 2h and Fig. S1f). Some parts of Eastern Europe bordering Russia are exceptional (Fig. 2e), where there is increase in total simulated NPP.

In response to the instantaneous land-use change due to halving N, the temporal evolution of total annual NPP increases in the case of AROPAj and stabilizes within 4 to 6 years (Fig. S2a). In contrast, with the NLU scenario, at the beginning of the simulation years there is inter-annual variability (decrease in some years and increase in others) with negligible change in total NPP over time until the year 20 (Fig. S2a). By the end of 150 simulation years, we find a considerable decrease in NPP, however, the decrease is negligible when compared to the AROPAj scenario.

### Changes in biomass and soil carbon stock

With the AROPAj land-use scenario, the biomass C and soil C responses follow the NPP response (Fig. S2b, c). In response to the instantaneous land-use change due to halving N policy, the European ecosystems’ biomass and soils start sequestering C over time, stabilizing after around 150 years in our equilibrium simulations (Fig. S2b, c). At the beginning of the simulation, around 10 years, the annual mean total soil C sequestration is about 100 MtC (Fig. S2c). This increases steadily to stabilize at around 150 years with the total C sequestration in soils reaching more than 1000 MtC (Fig. S2c). Thus at the whole EU scale we find an increase in total soil C of 1014 MtC (Tables 3, 4). More than 50% of this soil C sequestration occurs in Germany (120.23 MtC), France (122.86 MtC), Italy (103.71 MtC), Poland (137.38 MtC) and Romania (108.99 MtC) (Tables 3, 4 and Fig. S3a).

For the NLU scenario, following the instantaneous land-use change due to halving N policy, the EU ecosystem biomass and soils experience a reduction in C sequestration over time, stabilizing after around 100 years in our equilibrium simulations (Fig. S2b, c). At the beginning of the simulations, around 10 years, the reduction in soil C sequestration is about 9 MtC (Fig. S2c). This steadily decreases to stabilize after around 100 years with total soil C reduction reaching ten times the initial reduction (97 MtC, Fig. S2c). Among EU countries the major decline in soil C sequestration occurs in Spain (− 12.64 MtC), France (− 14.22), Germany (− 6.82 MtC), Poland (− 7.83 MtC) and Romania (− 8.73 MtC) (Tables 3, 4 and Fig. S3e). These are the countries which experience a large decline in grasslands and pasture lands and an increase in cropland areas.

### Changes in biodiversity

**Figure 3:**
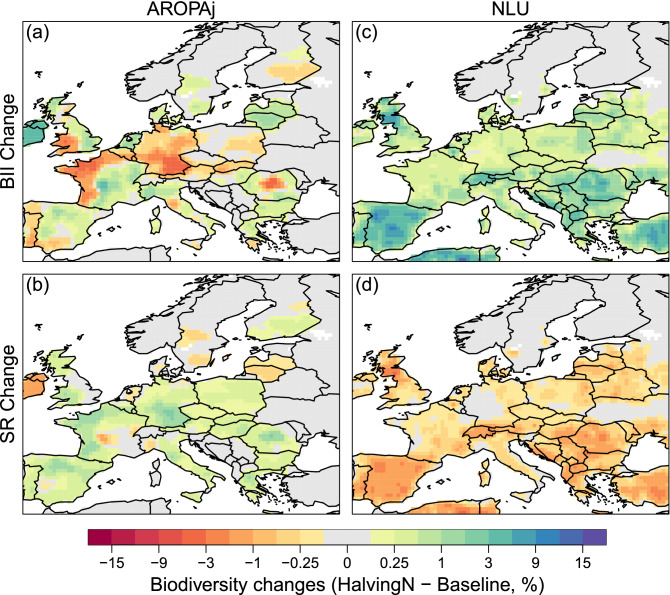
Biodiversity Intactness Index (BII) and Species Richness (SR) changes across the EU as computed by the PREDICTS models for the AROPAj (**a**, **b**) and NLU (**c**, **d**) land-use change scenarios. The changes are calculated as differences between the *Halving-N* and *Baseline* simulations. BII indicates average abundance of a taxonomically and ecologically broad set of species in an area relative to their abundances in an intact reference ecosystem. The SR reports the number of species, relative to the number expected in a natural system. This figure is created using software R version 3.6.0 (https://www.r-project.org).

Here we assess two biodiversity change indicators (BII and SR) from the PREDICTS model output. With the AROPAj land-use scenario, the PREDICTS models simulate an increase in both BII and SR (spatial mean change across EU respectively 2.0 and 1.9%) due to halving N fertilizer (Fig. 3a, b). In AROPAj, the increase in forest, pasture and other herbaceous vegetation areas at the expense of cropland leads to an increase in the number of species (increase in SR). With the NLU land-use scenario, PREDICTS simulates on average a small increase in BII and a small decrease in the relative number of species (spatial mean change across EU respectively + 1 and − 0.4%) in the *Halving-N* scenario compared to the *Baseline* (Fig. 3c, d). The decrease in SR due to the decrease in cropland yield is partially offset by the replacement of pasture (an ecosystem with high species richness) by cropland (an ecosystem with lower species richness). Moreover, replacement of pastureland ecological communities (very different to the ones found in primary natural ecosystem) by cropland ecological communities (equivalent to those found in the primary ecosystem) leads to the ecological communities more similar to the ones found in the primary ecosystem (expressed in the following as more naturalness of the ecosystem) which is simulated through increased BII.

### Spatial comparison of carbon and biodiversity changes

**Figure 4:**
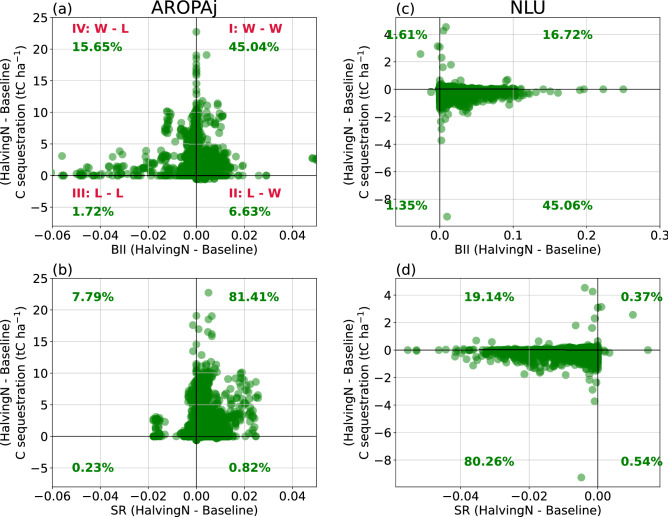
The distribution of spatial grid points across four quadrants described here for AROPAj (**a**, **b**) and NLU (**c**, **d**) scenarios. Panels (**a**) and (**c**) describe the benefits in terms of carbon (C) sequestration and Biodiversity Intactness indicator (BII). Panels (**b**) and (**d**) describe the benefits in terms of C sequestration and Species Richness (SR). Y-axis is the change in C sequestration between *Halving-N* and *Baseline* at each grid point across the EU as simulated by ORCHIDEE-crop. X-axis is the change in BII (**a**, **c**) and SR (**b**, **d**) between *Halving-N* and *Baseline* at each grid point across the EU as computed by the PREDICTS model. The text in the panel (**a**): I quadrant “W–W” refers to Win–Win situation (+ ve change in C sequestration and BII), II quadrant “L–W” refers to “Loss–Win” situation (− ve change in C sequestration while + ve change in BII), III quadrant “L–L” refers to “Loss–Loss” situation (− ve change in both C sequestration and BII), IV quadrant “W–L” refers to “Win–Loss” situation (+ ve change in C sequestration and –ve change in BII). The % change numbers in each quadrant represents the % of total grid points across EU. This figure is created using community data analysis tools with Python version 3.7.10 (CDAT (https://cdat.llnl.gov/)).

We quantify the benefits in terms of soil C sequestration and biodiversity indicators by identifying the distribution of grid points across four quadrants (Fig. 4). For the AROPAj land-use scenario, we find that 45% of ecosystem grid points experience positive change (see quadrant I of Fig. 4a). We refer to this as a Win–Win situation, i.e. those 45% grid points experience an increase in soil C sequestration and more naturalness in the composition of ecological communities (increase of BII). Fewer than 2% of the total grid cells experience loss in both ecosystem C and BII (Loss- Loss situation). The remaining 22% grid cells experience counteracting responses in terms of ecosystem C and BII (i.e. quadrants II and IV, Win–Loss situation). All the EU countries analyzed here experience Win–Win situations in terms of soil C sequestration and BII (Table 3).

We also find a similar response in terms of changes in soil C sequestration and SR for the AROPAj land-use scenario (Fig. 4b). Nearly 81% of all grid cells experience a Win–Win situation (see soil carbon vs SR Fig. 4b, quadrant I), less than 1% fall within quadrant III (Loss–Loss situation), 9% fall within quadrants II and IV (Win–Loss and Loss–Win situations) and the remaining 9% of grid cells experience no change and hence do not fall within any quadrants.

With the NLU scenario, we find ~ 80% of all grid cells experience a Loss-Loss situation in ecosystem C and SR (Fig. 4d). This reflects in most countries (Table 3). However, we find a difference in response for soil C vs BII when compared with the AROPAj scenario. In NLU, 45% of all grid cells experience a Loss-Win situation in terms of ecosystem C and BII (Fig. 4c). That is those grid cells fall within quadrant IV where soil C sequestration is negative (carbon loss) and BII positive (improvement in the composition of ecological communities). Fewer than 2% of grid cells experience loss in both C sequestration and BII. Furthermore, fewer than 2% of grid cells experience a counteracting carbon and BII change. Nearly 17% of the grid cells experience a Win–Win situation. Due to the loss of pasture and other herbaceous vegetation with the increase in cropland area, the ecosystem experiences a loss in C sequestration capacity and the naturalness of ecological communities improves despite a decrease in species richness.

### Breakdown of changes for carbon and biodiversity

**Figure 5:**
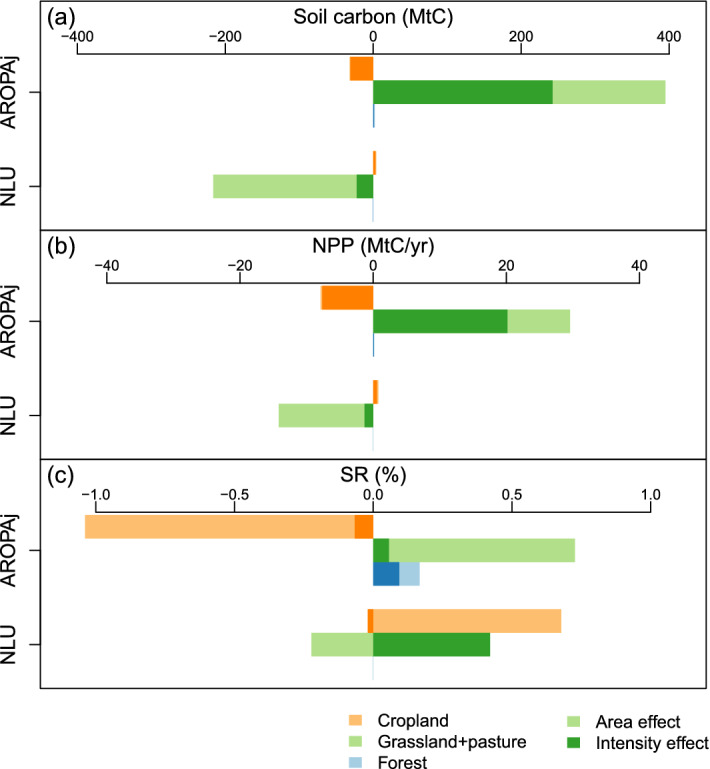
Breakdown of (**a**) soil carbon, (**b**) net primary production (NPP), and (**c**) species richness (SR) change from present-day (*Baseline*) due to “area effect” and “intensity effect” at the EU scale. Colors (orange, green and blue) distinguish the different land-uses (cropland, grassland/pasture and forest). Dark color shows the “intensity effect” and light color shows the “area effect”. Breakdown of changes (*Halving-N*–*Baseline*) are computed for both AROPAj and NLU scenarios. The mean numbers are given in Tables S1 and S2. The “area effect” means the change in C sequestration/NPP/SR associated with a change in the land-use area and the “Intensity effect” means the effect of production reallocation on C sequestration/NPP, and the effect of a change in land-use intensity (crop yield or stock rate) on biodiversity. This figure is created using software R version 3.6.0 (https://www.r-project.org).

In this section, we present the results from the break-down method discussed in “Breakdown method for biodiversity and carbon changes” section above. The break-down method is applied for the changes between the *Halving-N* scenario and the *Baseline* scenario. This break down shows the changes associated with the change in the intensity of the environmental indicator (“Intensity effect”) and in area (“Area effect”) for each land-use at the EU scale (Fig. 5). As described in the methods “Breakdown method for biodiversity and carbon changes” section, the “Area effect” corresponds to the change in the environmental indicator associated with a change in the land-use area and the “Intensity effect” corresponds to the effect of production reallocation on C sequestration, and the effect of a change in land-use intensity (crop yield or stock rate) on biodiversity.

In the AROPAj land-use scenario, we observe an overall higher C sequestration (+ 365 MtC for soil carbon and + 22 MtC/yr for NPP, Fig. 5a) and a similar species richness level (− 1%) in the *Halving-N* scenario compared to the *Baseline* scenario (Fig. 5c). The higher C sequestration in the *Halving-N* scenario compared to the *Baseline* scenario occurs mainly in grassland soils (+ 395 MtC) and in grassland NPP (+ 30 MtC). This higher C sequestration is partially offset by CO_2_ emissions from cropland soils (− 32MtC) and NPP (− 8MtC) (Fig. 5a). Differences in forest environmental indicators are small for AROPAj, because of the small difference in forest area between scenarios in the EU.

The higher C sequestration in grassland soils in the *Halving-N* scenario is due to a larger grassland area (+ 153 MtC, area effect) and a higher C sequestration (leading to an increase of carbon sequestration of + 243 MtC on the overall grassland area, Intensity effect) in the EU (Fig. 5a). This grassland area increase (+ 1.8 Mha) is due to an extensification of livestock production with a decrease in the livestock stocking rate (− 0.1 heads/ha) in line with the reduction in livestock production (− 1.6 Mheads) in the EU (Table S1). For vegetation, C sequestration follows the trends in soil C with a smaller amplitude. The higher C sequestration per hectare in the *Halving-N* scenario compared to the *Baseline* scenario results from an expansion of grassland on land with high C sequestration rates.

In the AROPAj land-use scenario, the similar SR levels in the *Halving-N* scenario and in the *Baseline* scenario (− 0.1%) are due to the offset of biodiversity losses in cropland areas (− 1%) by the increase of biodiversity in pasture areas (+ 0.7%) (Fig. 5b). This lower level of SR in the *Halving-N* scenario compared to the *Baseline* scenario is here due to the agricultural abandonment represented in AROPAj which leads to a reduction in the area under cultivation in favor of fallow. The expansion of fallow leads to lower species richness as fallow land considered in the biodiversity models has a minimum intensity annual crop (see Table 1) and with lower biodiversity levels per unit area than the light intensity cropland class of the PREDICTS models (See coefficients of species richness in PREDICTS models^45^).

In the NLU land use scenario, we observe an overall lower C sequestration (− 212 MtC for soil C and − 13 MtC/yr for NPP) and a similar species richness level (+ 0.5%) in the *Halving-N* scenario compared to the *Baseline* scenario (Fig. 5a). The lower C sequestration occurs mainly in grassland soils (− 216MtC) and in grassland NPP (− 15MtC). This is due to a decline in grazed areas (− 194MtC for soil carbon and − 13MtC/yr for NPP). For croplands, the dynamics of carbon in soils and in NPP between *Halving-N* and *Baseline* has a negligible effect on the overall carbon balance (the carbon sequestration in cropland soil is + 4MtC, see Fig. 5a). The negligible effect in cropland is due to lower crop yield in the *Halving-N* scenario than in *Baseline* (− 0.55 tDM/ha) which leads to a lower EU crop production (− 0.2 Pkcal), despite a higher cropland area (+ 5Mha) in the *Halving-N* scenario than in *Baseline* (see Lungarska et al.^36^ for an economic explanation of this “extensification” mechanism). However, cropland extensification leads to an increase in C sequestration with an increase in land area but is to a large extent part offset by the lower EU crop yields in the *Halving-N* scenario compared to the *Baseline* (Fig. 5c).

In the NLU land-use scenarios, the similar SR across all land-uses (+ 0.5%) in the *Baseline* and *Halving-N* scenarios is actually the result of contrasting SR dynamics in cropland and grassland areas. The biodiversity levels are higher in cropland in the *Halving-N* scenario compared to the *Baseline* mainly due to larger cropland area (+ 0.7%). On the contrary, the species richness is smaller in the *Halving-N* scenario compared to the *Baseline* because a lower crop yield (− 0.4%) offsets the lower biodiversity levels associated with a reduction in grassland (− 0.6%) in the *Halving-N* scenario compared to the associated *Baseline* scenario.

## Discussion and conclusions

This study investigates the benefits on ecosystem C and biodiversity of a policy scenario reducing mineral N fertilizer use by 50% from present-day levels across EU agriculture (Fig. 1). Applying the 50% N-fertilizer-reduction policy to the AROPAj and NLU economic models produces land-use changes (see Fig. S1). These land-use changes were provided as input to the ORCHIDEE-crop and PREDICTS models.

We find a contrasting response in both ecosystem C and biodiversity indicators between the AROPAj and NLU land-use change scenarios (Figs. 2, 3, 4, 5) highlighting the structural dependence of the results on the economic models used in this study. The scenarios produced by the two economic models correspond to two different ways of implementing nitrogen reduction scenarios: a massive land abandonment with a large reduction in agricultural production (AROPAj); an extensification of crop production with a smaller reduction in agricultural production (NLU). The land abandonment scenario leads to higher C levels in soil and in biomass, and similar species richness levels compared to the *Baseline*. On the contrary, the scenario of more extensive crop production leads to the expansion of cropland area to the detriment of pasture in the NLU *Halving-N* scenario compared to the *Baseline*. This leads to lower carbon levels, especially in soil, and similar species richness levels.

The similar species richness levels in the AROPAj and NLU land-use scenarios actually conceal two different mechanisms that strongly impact biodiversity. In the AROPAj land-use scenario, land abandonment leads to lower biodiversity levels in cropland as they are more intensively managed in the *Halving-N* scenario and a higher biodiversity level in grassland areas. But the biodiversity loss described in crops is probably overestimated because of how fallow is represented in the modelling framework of this study. Here, fallow is considered as a zero-yield annual crop (as is the case in this study), the conversion of crops to this land-use leads to a reduction in species richness in the PREDICTS models. But there are contexts where this conversion may lead to biodiversity gains that are not considered in this modelling framework. Indeed, fallow land could be a transitional land use allowing the development or the implementation of alternative agricultural practices (e.g. organic farming, modeled neither by AROPAj nor by NLU) or other land uses such as forest. Furthermore, with steering from complementary policy tools such as payments for ecosystem services or for carbon storage in soils, these areas can provide valuable help in biodiversity restoration and climate change mitigation. On the contrary, the cropland expansion in NLU land-use scenario leads to higher biodiversity levels in cropland due to lower yields in the *Halving-N* scenario compared to the *Baseline*, offset partially by lower biodiversity levels in grassland.

Conversion of larger areas of fallow land and leguminous crops to productive natural grasslands in ORCHIDEE-crop for the AROPAj land-use scenario contributed to large C sequestration in soils. This is consistent with studies that show a positive assessment of restoring fallow land for the production of biomass for non-agricultural purposes^49,50^. Leguminous plants are known to contribute to ecosystem benefits such as increasing C sequestration in soils^51^. However, realistic representation of fallow land and specific crop types (e.g. leguminous crops, wheat, maize, etc.) in the ORCHIDEE-crop model is necessary to more accurately simulate land-use change impacts. In this study, we have only considered c3 winter, c3 summer and c4 summer crop types, c3/c4 natural grasslands and forests. In addition, how the model handles the Carbon and Nitrogen cycle processes and its interactions with biomass and soil carbon is important. The ORCHIDEE-crop version used in this study simplifies N fertilizer representation (uniform N-fertilizer application over croplands), hence more realistic representation of spatial variation of N-fertilizer application could provide improved spatial simulation of NPP and soil and biomass carbon. NPP reflects the carbon assimilated by the vegetation through photosynthesis that is available for allocation to biomass after accounting for autotrophic respiration. An increase in NPP permits the allocation of carbon for new leaves, roots and stems that could lead to an increase in biomass and sequester carbon in soils (Figs. S2, S3)^52^.

Despite the similarities with low intensification strategies like organic agriculture or agroecology, the *Halving-N* scenario represents only one aspect, which is a decrease in mineral N fertilizer input. In organic agriculture or agroecology, many other practices are combined to avoid substituting the effects of N fertilizer, like an increase in leguminous plants in rotation^53^ or increase in manure use. The first substitution is not represented in NLU, and the substitution of mineral fertilizer by manure is not possible in NLU and AROPAj because the higher feed price leads to lower animal production^29–31^ in the EU in the *Halving-N* scenario compared to the *Baseline*. Moreover, biodiversity levels are probably under-estimated as neither the amount of natural vegetation in agricultural landscapes^54^ nor pesticide levels are represented in this study, thus under-estimating the benefits of low-input systems such as organic agriculture compared to the *Halving-N* scenario.

The difference in C sequestration between the *Halving-N* scenario and the *Baseline* is estimated at between 368MtC (AROPAj land-use scenario) and − 225 MtC (NLU land-use scenario). The soil C pool constitutes about two-thirds of the total terrestrial C pool, which is three times the quantity of atmospheric carbon^55^. Thus, it is important to understand the changes in total soil C stocks across the EU due to land use/land cover changes induced by N fertilizer policy impacts. In the EU regulation scheme, the EU sets out the overall Union-wide target of net greenhouse gas removal in the land use, land-use change and forestry sector (LULUCF) sector at 310 million tons of CO_2_ (European Commission 2021). Taking land out of cultivation as represented in the AROPAj land-use scenario can contribute to this objective of net greenhouse gas removals in the LULUCF sector. Therefore, from a purely environmental point of view, our results suggest favoring land abandonment over extensification of production. However, this result raises major questions about the practical implementation of such an orientation, given its potentially significant economic and social consequences.

## Supplementary Information

Supplementary Information.

## Data Availability

The datasets presented in this study could be made available for downloading upon reasonable request to the corresponding author.

## Code availability

All the Python and R codes used to create figures are available upon reasonable request from the corresponding author. The *glmer* function of the *lme4* package of R was used to estimate the PREDICTS model functions. R was used to estimate the PREDICTS models and then python was used to use the models in a predictive way in the different cells of the gridded land use map from the land use models. R version 3.6.0 (https://www.r-project.org) and Python version 3.7.10 (https://www.python.org/) is used.
