# Supplementary material for: Quantifying the benefits of reducing synthetic nitrogen application policy on ecosystem carbon sequestration and biodiversity
Source: Sci Rep. 2022 Dec 1;12:20715. doi: 10.1038/s41598-022-24794-2 (PMC9715672; doi:10.1038/s41598-022-24794-2)
Supplement: Supplementary file 1 — Supplementary Information. [file 41598_2022_24794_MOESM1_ESM.pdf]

Supplementary Figures and Tables

Quantifying the benefits of reducing synthetic nitrogen application policy on ecosystem carbon sequestration and biodiversity

N. Devaraju[1], Rémi Prudhomme[2], Anna Lungarska[3], Xuhui Wang[4], Zun Yin[1], Nathalie de Noblet-Ducoudré[1], Raja Chakir[5], Pierre-Alain Jayet[5], Thierry Brunelle[2], Nicolas Viovy[1] , Adriana De Palma[6] , Ricardo Gonzalez[6,7] and Philippe Ciais[1]*

[1]Laboratoire des Sciences du Climat et de l`Environnement LSCE/IPSL, Unité mixte CEA-CNRS-UVSQ, Université Paris-Saclay, F-91191 Gif-sur-Yvette, France.
[2]Cirad, UMR CIRED, 94736 Nogent-sur-Marne, France.
[3]US ODR, INRAE, 31326 Castanet-Tolosan, France.
[4]Peking University, College of Urban and Environmental Sciences, Beijing, China.
[5]Université Paris-Saclay, INRAE, AgroParisTech, PSAE, F-91120, Palaiseau, France.
[6]Department of Life Sciences, Natural History Museum, Cromwell Road, London SW7 5BD, United Kingdom.
[7]Department of Life Sciences, Imperial College London, Silwood Park, Berkshire SL5 7PY, United Kingdom
**Currently at Centre for Biogeochemistry and Anthropocene and Department of Geosciences, University of Oslo, Oslo, Norway.*

Corresponding author:
Narayanappa Devaraju
devarajun@gmail.com

Figure S1: Land cover and land-use changes described by changes in the area of plant functional types of ORCHIDEE-CROP between *Halving-N* scenario and *Baseline* scenario. Economic land use models AROPAj and NLU have computed land use changes, which are used as input to ORCHIDEE-CROP and PREDICTS models. This figure is created using NCAR commanding language version 6.6.2 (NCAR Command Language (NCL) (ucar.edu)).

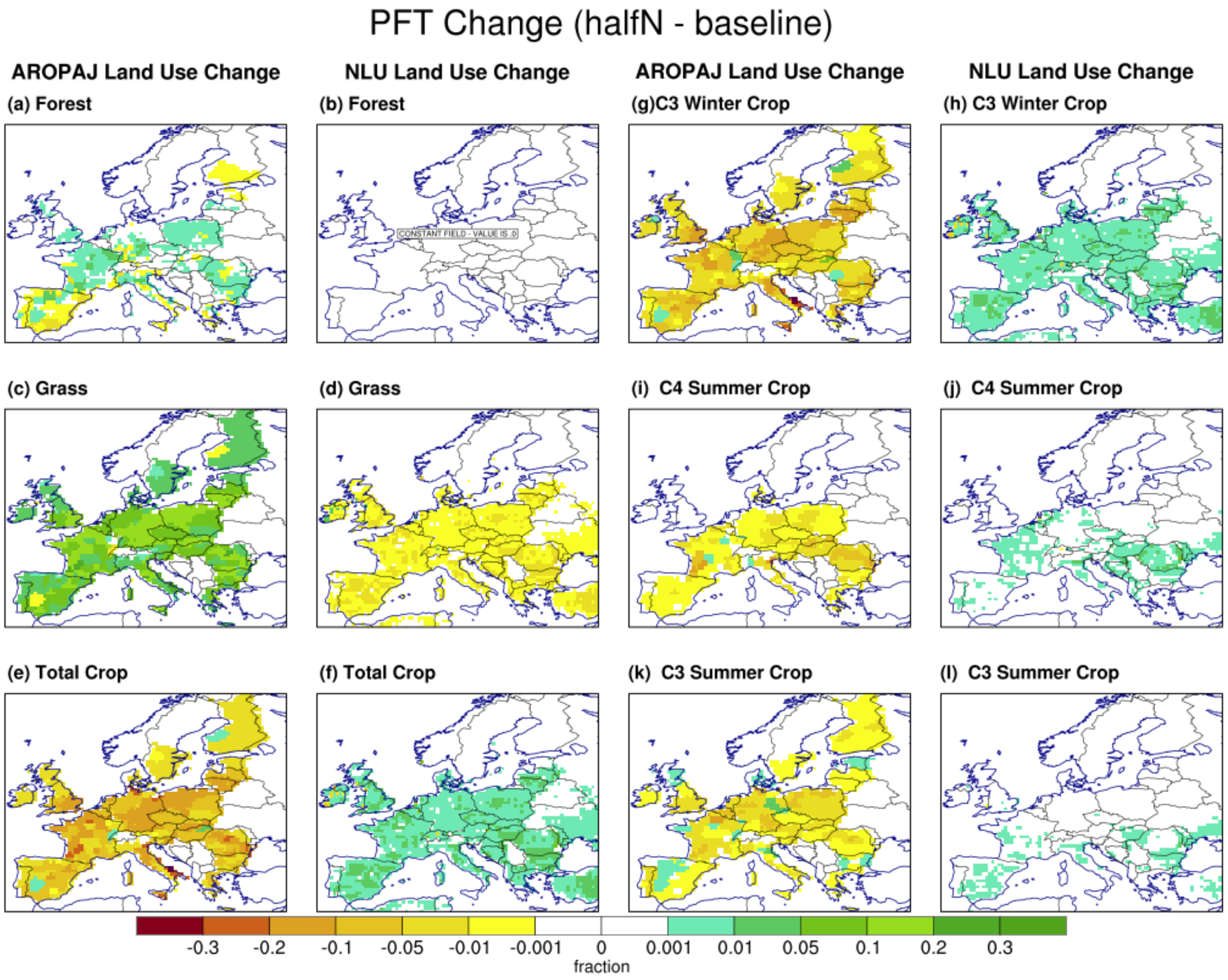

Figure S2: Annual total changes in (a) Net Primary Production (NPP), (b) Biomass carbon, and (c) Soil carbon simulated by ORCHIDEE-CROP model. The changes are computed as the difference between the *Halving-N* and *Baseline* simulations for both AROPAj and NLU land use scenario cases. For all variables, a five-year moving average is applied to original annual mean data. This figure is created using community data analysis tools with Python version 3.7.10 ([CDAT (llnl.gov)](https://cdat.llnl.gov))

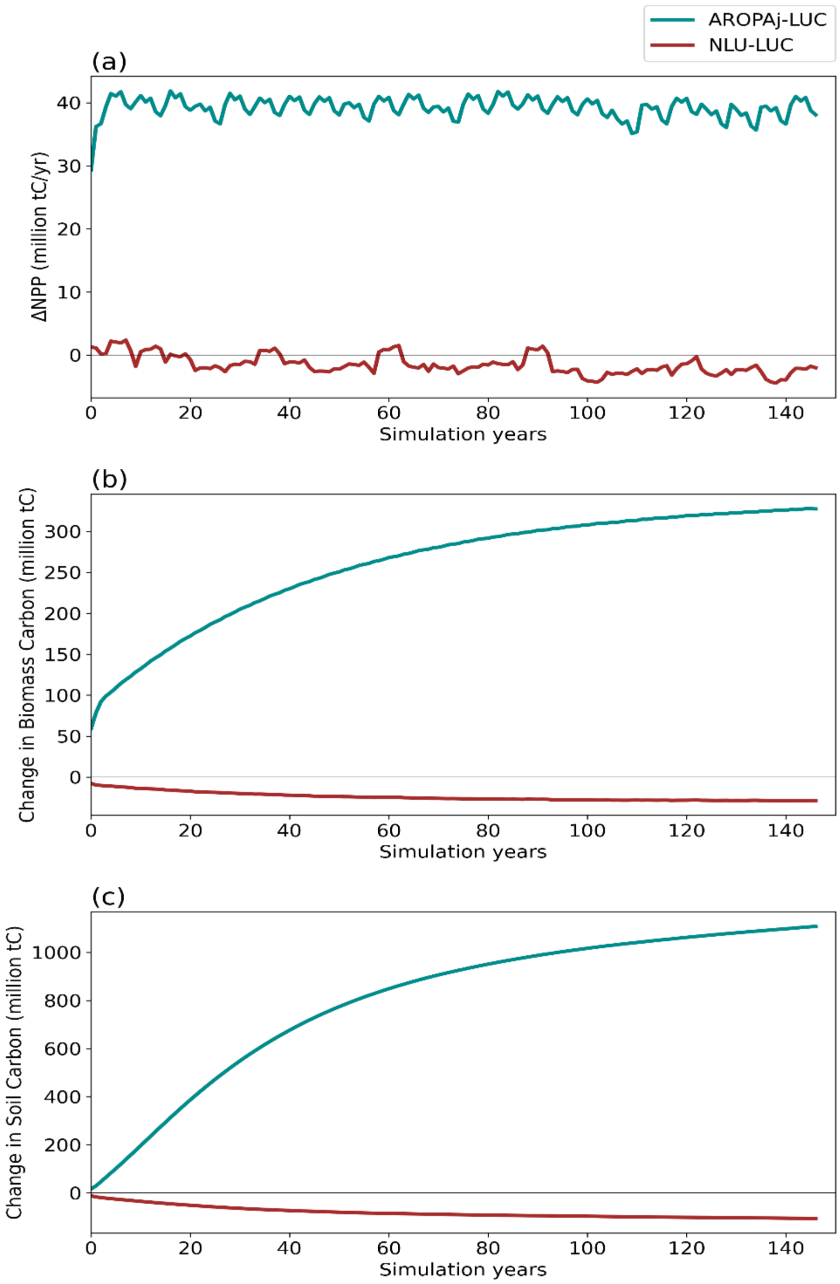

Figure S3: ORCHIDEE-CROP model simulated annual mean change in (a, e) total soil carbon (tC ha^{-1}), (b, f) forest soil carbon, (c, g) grass and pasture soil carbon, (d, h) crops soil carbon due to 50% reduction in N fertilizer. The mean changes are computed using the last 50 years' means of the 150-year simulations. Change in total soil carbon shown here is the weighted sum across PFT types. Stippled areas are regions where changes are statistically significant at the 95% confidence level. Significance level is estimated using a Student's t-test with a sample of 50 annual mean differences and standard error corrected for temporal serial correlation. This figure is created using NCAR commanding language version 6.6.2 (NCAR Command Language (NCL) (ucar.edu)).

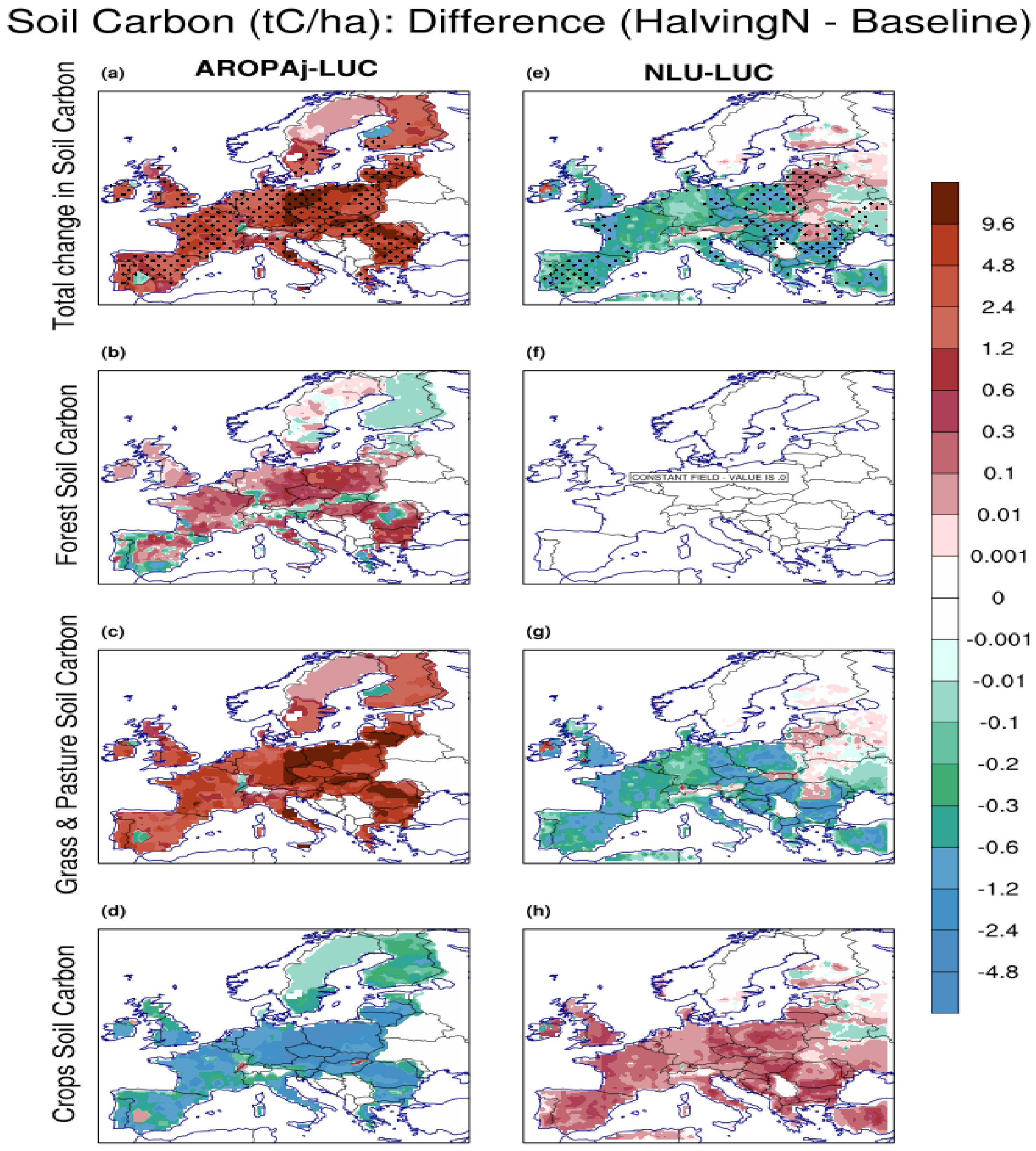

Table S1: Variables used in the breakdown to explain the environmental changes in the scenario of *Halving-N* consumption, in the *Baseline* and the difference between *Halving-N* and *Baseline* in forest, pasture + grassland and cropland in EU.

		**Value in *Baseline***		**Value in *Halving-N***		**Change between *Halving-N* and *Baseline***	
		AROPAJ	NLU	AROPAJ	NLU	AROPAJ	NLU
**Cropland**	Area (Mha)	115,1	131,6	110,8	136,6	-4,3	5,0
	Production (MtDMl)	397,8	378,2	264,2	316,3	-133,6	-61,9
	Yield (Mkcal/ha)	3,5	2,9	2,4	2,3	-1,1	-0,6
**Pasture + Grassland**	Area (Mha)	51,5	65,1	53,3	60,1	1,9	-5,0
	Production (000 head)	128,5	47,0	126,9	44,3	-1,6	-2,7
	Yield (000 head/Mha)	2,5	0,7	2,4	0,7	-0,1	0
**Forest**	Area (Mha)	161,8	225,0	162,3	225,0	0,5	0
	Production (000 head)	-**	-**	-**	-**	-**	-**
	Yield	-**	-**	-**	-**	-**	-**

** Not represented in AROPAJ and NLU

Table S2: Breakdown of carbon sequestration and biodiversity changes between a scenario of *Halving-N* consumption compared to a *Baseline* scenario based on the Area effect, intensity effect and total effect for land-uses forest, pasture plus grassland and cropland in EU.

		Effect on soil carbon (MtC)		Effect on NPP (MtC)		Effect on SR (%)	
		AROPAJ	NLU	AROPAJ	NLU	AROPAJ	NLU
Cropland	Total	-32,0	4,0	-7,9	0,8	-1	0,7
	Intensity	-30,9	3,0	-7,6	0,5	-0,1	0
	Area	-1,1	1,1	-0,3	0,2	-1	0,7
Pasture + Grassland	Total	395,0	-216,2	29,6	-14,2	0,7	-0,2
	Intensity	242,5	-22,4	20,2	-1,3	0,1	0,4
	Area	152,5	-193,9	9,4	-12,9	0,7	-0,6
Forest	Total	2,0	-0,2	0,1	0,0	0,2	0
	Intensity	1,2	-0,2	0,1	0,0	0,1	0
	Area	0,8	0,0	0,1	0,0	0,1	0

* The effect represented here is the "yield offset" which corresponds to the avoided land-use change allowed by a yield change (See the Methods section for more information)
